# Effect of Vitreous Reflux after Intravitreal Aflibercept Injection for Macular Edema with Branch Retinal Vein Occlusion: A Real-World Study

**DOI:** 10.1155/2024/7645490

**Published:** 2024-06-25

**Authors:** Tetsuya Muto, Masaaki Sakamoto, Shigeki Machida, Shinichiro Imaizumi, Tetsuju Sekiryu

**Affiliations:** ^1^ Department of Ophthalmology Dokkyo Medical University Saitama Medical Center, Koshigaya, Japan; ^2^ Imaizumi Eye Hospital, Koriyama, Japan; ^3^ Department of Ophthalmology Fukushima Medical University, Fukushima, Japan

## Abstract

**Purpose:**

This study aimed to evaluate the therapeutic effect of vitreous reflux (VR) after intravitreal aflibercept injection (IVAI) for macular edema (ME) following naïve branch retinal vein occlusion (BRVO).

**Methods:**

Eighty patients with ME following BRVO were divided into three groups according to the conjunctival bleb diameter after IVAI as follows: group A (no VR), group; B (<3 mm VR), and group C (>3 mm VR). Each patient received single IVAI. The treatment response was evaluated with the best-corrected visual acuity (BCVA) and optical coherence tomography measurements of the retinal foveal thickness (RFT) before treatment and 1 month after the first injection. RFT >375 *μ*m was defined as recurrence and received additional IVAI. The recurrence rate of ME and total numbers of IVAI were investigated at 12 months.

**Results:**

The BCVA values at 1 month were 0.17 ± 0.29 in group A (*n* = 41), 0.18 ± 0.17 in group B (*n* = 18), and 0.19 ± 0.26 in group C (*n* = 21). The RFT at 1 month were 270 ± 45 *μ*m in group A, 279 ± 24 *μ*m in group B, and 290 ± 43 *μ*m in group C, respectively. ME recurred in 29 out of 41 patients in group A, 15 out of 18 in group B, and 14 out of 21 in group C. The total numbers of IVAI were 2.50 ± 1.24 in group A, 2.59 ± 1.06 in group B, and 2.29 ± 1.27 in group C, respectively. In the above mentioned comparisons, no significant differences were found following an IVAI (*P* > 0.05).

**Conclusions:**

VR after IVAI did not affect the therapeutic effect in patients with ME following BRVO. Thus, we do not need to pay excess attention to VR in the case of IVAI.

## 1. Introduction

Intravitreal anti-vascular endothelial growth factor (anti-VEGF) agents are the current standard of care for many retinal diseases [1]. Because of repeated injections in the same disease, the number of cases will increase progressively. Vitreous reflux (VR) often occurs in the case of intravitreal anti-VEGF agent injection [2–4]. Once VR occurs, the risk of complications such as retinal detachment, endophthalmitis, and cystoid macular edema (ME) due to vitreous incarceration may increase. Given that VR may include the injected agents, the effect on retinal diseases may be reduced. Essentially, VR included the injected agents using rabbit eyes [5] and cadaver eyes [6].

Several studies have reported a small scheme for the needles [7, 8], injection methods [9–12], and injection sites [13] to reduce VR. However, such a small scheme cannot get rid of VR completely. Thus, the effect of VR must be investigated in clinical practice.

In investigating the effect of VR on injection agents, selecting conditions as a target disease that has received intravitreal injections several times is difficult. Uyar et al. reported that the number of previous intravitreal ranibizumab injections negatively correlated with the VR rate [3]. Accuracy may be worse than non-previous intravitreal injection cases.

Cases of naïve ME following branch retinal vein occlusion (BRVO) cases were adopted in the current report. The selected patients have no previous intravitreal injection, subtenon injection, or oral administration of carbonic anhydrase inhibitor. Such cases received intravitreal aflibercept (Eylea®; Regeneron, Tarrytown, NY, USA) injection (IVAI), and the effect of VR on treatment was investigated.

## 2. Materials and Methods

The study adhered to the tenets of the Declaration of Helsinki. Its protocol was approved by the Institutional Review Board at Dokkyo Medical University Saitama Medical Center (approval no. 202202). In total, 80 eyes of 80 patients with ME complicated by BRVO who visited Dokkyo Medical University Saitama Medical Center between February 2017 and November 2022 were evaluated. Patients were randomly selected.

**Figure 1:**
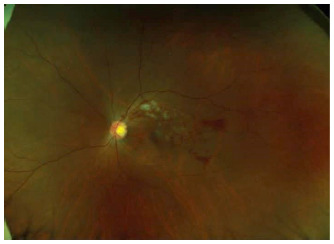
A representative photo showing a retinal hemorrhage in the flow region of the superotemporal vein in the left eye of a 38-year-old woman.

At the initial visit, all patients underwent comprehensive ophthalmologic evaluations, including standardized refraction and measurement of the best-corrected visual acuity (BCVA) using a Landolt ring, slit-lamp biomicroscopy, axial length measurement, optical coherence tomography (OCT), and color fundus photography (Figure 1(a)). The BCVA represents a logarithm of the minimal angle of resolution (logMAR). Ophthalmologic evaluations, including the measurement of BCVA, slit-lamp biomicroscopy, and OCT, were performed every month.

**Figure 2:**
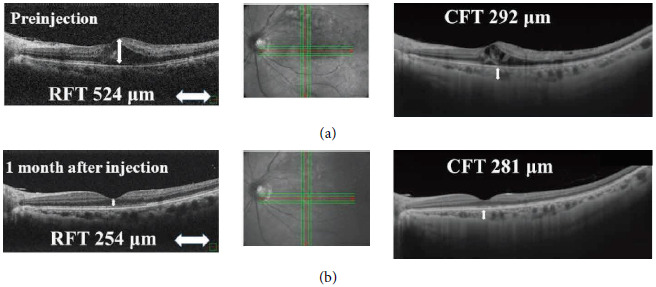
Optical coherence tomography (OCT) scans suggest the presence of macular edema in the left eye at the initial visit. The white arrows indicate the scanning direction. Retinal foveal thickness (RFT) was 524 *μ*m and choroidal foveal thickness (CFT) was 292 *μ*m (a). OCT scans showing improved macular edema 1 month after intravitreal aflibercept injection in the left eye. RFT reduced dramatically. Conversely, CFT remained almost unchanged (b).

All patients had an axial length measured by IOLMaster 700 (Carl Zeiss Meditec AG, Jena, Germany). Retinal morphology was assessed using spectral-domain OCT (RS-3000 Advance, Nidek Corporation, Japan) (Figure 2). OCT scans passed through the fovea horizontally and vertically. The averaged retinal foveal thickness (RFT) was determined by averaging the horizontal and vertical thicknesses at the fovea. In addition, swept-source OCT (PLEX Elite 9000, Carl Zeiss Meditec AG, Jena, Germany) was performed to determine the choroidal foveal thickness (CFT) by manually measuring the distance from the outer border of the retinal pigment epithelium to the inner border of the sclera at the fovea (Figure 1(b)). One of the authors (M.S.) independently evaluated OCT images in a blinded fashion, not knowing the clinical information of any patients. Ophthalmologic evaluations, including BCVA measurement, slit-lamp biomicroscopy, and OCT, were performed every month. RFT >375 *μ*m was defined as recurrence, and such a case received additional IVAI (Figure 2).

Patients were considered for study participation if they met all the following inclusion criteria: symptomatic BRVO with retinal edema, foveal center involvement, foveal thickness >375 *μ*m at the initial study visit (measured by OCT), and symptom duration of less than 4 months before examination. The BRVO diagnosis was based on fundus examination and fluorescein angiography findings and was determined by one of the authors (T.M.). Eyes with hemicentral retinal vein occlusion were included, but eyes with central retinal vein occlusion were excluded from this study. Eyes were also excluded based on the following criteria: other chorioretinal diseases, such as diabetic retinopathy, hypertension retinopathy, retinal macroaneurysm, age-related macular degeneration, myopic choroidal neovascularization, uveitis, and retinitis pigmentosa, senile cataract that resulted in poor image quality, or any interventions for ME before the study period. Patients who had a history of intravitreal injection, vitrectomy, or any glaucoma surgery were excluded from this study. Pseudophakic patients who had undergone uncomplicated cataract surgery more than 3 months and had a history of retinal photocoagulation cases were included in this study. All patients did not use a Honan intraocular pressure reducer before intravitreal injection. All patients were treated with an IVAI (2 mg/0.05 mL) by one of the authors (T.M.) using a 32-gauge needle (Dentronics 32 G®; Dentronics, Tokyo, Japan) as described previously [2]. In an operating room under an operating microscope, IVAI was performed into the superotemporal quadrant of the right eye or superonasal quadrant of the left eye via the pars plana and into the vitreous cavity 3-4 mm posterior to the corneal limbus. After the injection, VR from the injection part was measured immediately using a caliper. The intraocular pressure (IOP) was measured immediately after IVAIs using an ICare PRO® (Tiolat, Helsinki, Finland). The immediate postinjection IOP was consistently measured within 1 min after the IVAIs. Patients were assigned to one of the three groups according to their VR diameter as follows: group A had no VR, group B had a VR diameter <3 mm, and group C had a VR diameter of >3 mm [3, 4]. Data obtained from the first session of the injections were used for this study.

Our usual BRVO treatment strategy is one initial IVAI followed by pro re nata.

Once RFT exceeds 375 *μ*m, additional IVAI will be performed.

All data are expressed as means ± standard deviations. To determine the statistical significance among the three groups, the data were analyzed using the Shapiro–Wilk test to check for normal distribution. In case of normal distribution, a one-way repeated-measures analysis of variance was employed. In case of abnormal distribution, the Kruskal–Wallis test was used. As post hoc tests, Bonferroni tests were also performed after the Kruskal–Wallis tests. The Chi-square tests were used among the three groups in sex, laterality, lens status, glaucoma, smoking condition, background systemic diseases, and recurrence rates. The statistical analysis was performed using StatMate version V for Macintosh (ATMS, Tokyo, Japan). A *P* value <0.05 was considered significant.

## 3. Results

Table 1 shows the background of the patients. No significant differences were observed among the three groups except for the axial length (*P*=0.025), IOP immediately after IVAI (*P*=0.0041), and HbA1c (*P*=0.029). Although significant differences were noted in the axial length and HbA1c using Kruskal–Wallis tests, no significant differences were detected in Bonferroni tests about the above mentioned two factors. A significant difference was observed in the IOP immediately after IVAI between group A and group C (*P* < 0.01) using Bonferroni tests.

**Figure 3:**
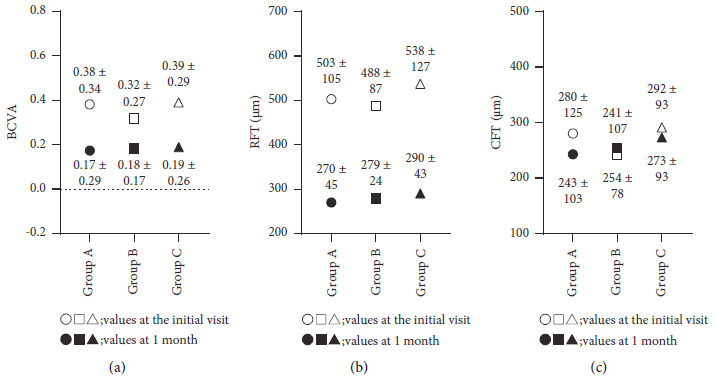
(a) No significant differences were observed in the best-corrected visual acuity (BCVA) at the initial visit (*P*=0.75). The BCVA was dramatically improved 1 month after intravitreal aflibercept injection and no significant differences were observed among the three groups (*P*=0.67). (b) The retinal foveal thickness was approximately 500 *μ*m at the initial visit, and no significant differences were observed among the three groups (*P*=0.43). The retinal foveal thickness was within 300 *μ*m 1 month after therapy, and no significant differences were noted among the three groups (*P*=0.19). (c) The choroidal foveal thickness at the initial visit was nearly the same among the three groups, and no significant differences were observed (*P*=0.48). The choroidal foveal thickness remained almost unchanged 1 month after therapy, and no significant differences were observed among the three groups (*P*=0.58).

No significant differences were noted in BCVA, RFT, and CFT at the initial visit among the three groups (Figures 3(a) and 3(c); *P*=0.75, *P*=0.43, and *P*=0.48).

No significant differences in BCVA, RFT, and CFT 1 month after IVAIs were found among the three groups (Figures 3(a)–3(c); *P*=0.67, *P*=0.19, and *P*=0.58).

**Figure 4:**
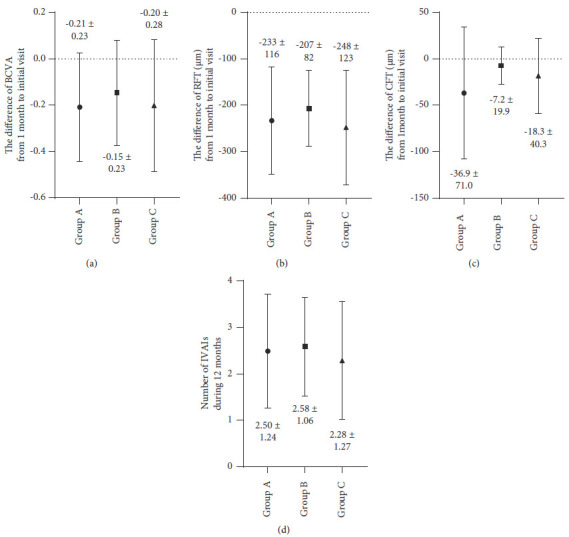
(a) The differences in best-corrected visual acuity from 1 month to the initial visit were almost the same level, and no significant differences were observed among the three groups (*P*=0.66). (b) The differences in retinal foveal thickness from 1 month to the initial visit were around −200 *μ*m, and no significant differences were noted among the three groups (*P*=0.53). (c) The differences in choroidal foveal thickness from 1 month to the initial visit were <−50 *μ*m, and no significant differences were observed among the three groups (*P*=0.39). (d) The total number of intravitreal aflibercept injections during 12 months was around 2.5, and no significant differences were noted among the three groups (*P*=0.72).

No significant differences in the differences of BCVA, RFT, and CFT from 1 month to the initial visit were found among the three groups (Figures 4(a)–4(c); *P*=0.66, *P*=0.53, and *P*=0.39). No significant difference in the number of IVAIs during 12 months was found among the three groups (Figure 4(d), *P*=0.72).

No significant differences in recurrence rates were noted among the three groups (Table 2).

## 4. Discussion

Brodie et al. injected 50 *μ*l of hematoxylin dye into cadaver eyes, and VR included 0.37 *μ*l of the average volume of dye [6]. This result should be considered a reference because the molecular weight and viscosity differ between aflibercept and hematoxylin dye. However, VR did not affect only RFT and CFT but also the recurrence rate and total number of IVAIs during 12 months. Because aflibercept may be few in VR, it did not affect the above mentioned factors. Kimura et al. reported a useful method for detecting aflibercept in aqueous humor using mass spectrometry [14]. If we can check the total amount of aflibercept easily and accurately, this method will be useful clinically. To the best of our knowledge, no previous studies have shown VR relating to poorer visual outcomes.

Non-responders should be considered in the case of age-related macular degeneration and diabetic ME as target diseases [15]. Shimura et al. reported that 17.6% of the non-responders were detected when injected with ranibizumab for diabetic ME [16]. In several retrospective studies, the non-responder rates of age-related macular degeneration for anti-VEGF agents are between 5.2% and 15% [17–19]. Some cases had difficulty determining whether therapeutic outcomes were unfavorable because of the VR or nonresponse to anti-VEGF agents. By contrast, retinal vein occlusion responds well to anti-VEGF agents [20, 21]. Furthermore, there are no reports of non-responders of ME following BRVO (source: PubMed; keywords: non-responder, ME, and BRVO; years: 2000–2023) worldwide. The accuracy of our study appears reliable because the selected cases are all naïve, and the target disease responds well to anti-VEGF agents. The novelty of this study is high because not only RFT and CFT 1 month after IVAI but also recurrent rates were investigated.

In the current study, VR occurred in nearly half of the cases, i.e., 39 out of 80 eyes. Pang et al. reported that the injection of intravitreal anti-VEGF agents (ranibizumab and aflibercept) using a 32-gauge needle and VR was noted in 4 out of 31 eyes [8]. Alshahrani et al. reported that intravitreal ranibizumab injections using a 32-gauge needle and VR were noted in 19 out of 86 eyes [22]. They did not adopt a special injection technique, such as a tunneled incision [8, 22]. Although they used the same 32-gauge needles, the reason for the difference remains unclear.

Our previous study [2] indicated that no significant difference in the frequency of VR was observed between patients injected using 30-gauge needles (38/116) and patients injected using 32-gauge needles (31/104, *P*=0.64). Furthermore, IOP immediately after injection was significantly higher in patients injected using 30-gauge needles than in patients injected using 32-gauge needles (*P* < 0.01). Taken together, we selected 32-gauge needles to avoid high IOP immediately after injection.

Significant differences were found in the axial length and HbA1c between groups A and B. According to our previous report [23], no correlation was found between the axial length and VR. Several studies have reported the correlation between axial length and IOP spike right after intravitreal injection [3, 12, 24–26]. Uyar et al. reported that the IOP spike right after intravitreal ranibizumab injection increased as the size of VR decreased [3]. Although we investigated IOP spikes using aflibercept, their result was almost the same as our result. To the best of our knowledge, no study has reported the correlation between VR and HbA1c. As the reason for the difference remains unclear, we will investigate this correlation next time.

Our ingenuity in this study included adopting patients with ME following naïve BRVO that did not have non-responders. Second, we integrated surgeon and needles. Furthermore, we gathered as many cases as possible and controlled confounders using the methods mentioned above.

This study has several limitations. As it is a real-world study, it does not provide a rationale for the sample size, so a lack of significant differences cannot be avoided. Future well-designed clinical studies that include a sample size rationale are needed. Second, we measured VR immediately after IVAIs only once and observed VR within 1 min after IVAIs. Measurement times and observation time might be insufficient. We could not determine whether VR measurement 1 or 3 h after IVAIs would make a difference in this study. In addition, we did not measure patients' scleral thickness which may be useful to share such a data.

## 5. Conclusions

Initial IVAIs were performed on patients with ME following naïve BRVO, and in the current study, the effect of VR was investigated. Although VR was observed in 39 out of 80 eyes, no effects were noted on the BCVA, RFT, and CFT, recurrence rates, and total numbers of IVAIs during 12 months. Taken together, VR after IVAI does not need excessive attention.

## Figures and Tables

**Table 1:** Clinical characteristics of eligible patients with macular edema following branch retinal vein occlusion.

	Group A	Group B	Group C	*P* value
Age (years)	64.4 ± 12.8	69.7 ± 7.5	60.5 ± 15.5	0.086
Sex (male, female)	19, 22	7, 11	12, 9	0.51
Laterality (right, left)	19, 22	13, 5	14, 7	0.11
Lens status (phakia, pseudophakia)	37, 4	16, 2	17, 4	0.61
From onset to initial visit (days)	38.9 ± 36.3	48.5 ± 35.9	55.9 ± 54.2	0.27
Axial length (mm)	23.88 ± 1.24	24.84 ± 1.54	24.57 ± 1.38	0.025
Spherical equivalent (diopter)	−1.00 ± 2.36	−2.00 ± 2.95	−2.04 ± 2.81	0.23
IOP immediately after IVAI (mmHg)	57.2 ± 10.4	51.7 ± 13.9	44.9 ± 13.9	0.0041
Glaucoma (yes, no)	2, 39	3, 15	3, 18	0.31
Smoking condition (smoker, nonsmoker, past smoker)	18, 11, 12	7, 2, 9	8, 8, 5	0.27
HbA1c (%)	5.6 ± 0.3	5.9 ± 0.4	5.8 ± 0.5	0.029
Hypertension (yes, no)	23, 18	9, 9	8, 13	0.41
Diabetes (yes, no)	3, 38	6, 12	3, 18	0.059
Hyperlipidemia (yes, no)	9, 32	8, 10	3, 18	0.077

Group A, no vitreous reflux; group B, vitreous reflux <3 mm; group C, vitreous reflux >3 mm.

**Table 2:** Recurrence rates in three groups through 12 months.

	Group A	Group B	Group C	*P* value
Rate of recurrence (yes, no)	29, 12	15, 3	14, 7	0.48

Group A, no vitreous reflux; group B, vitreous reflux <3 mm; group C, vitreous reflux >3 mm.

## Data Availability

The datasets used and/or analyzed during the current study are available from the corresponding author upon reasonable request.
