# Correction to “Effect of Vitreous Reflux after Intravitreal Aflibercept Injection for Macular Edema with Branch Retinal Vein Occlusion: A Real-World Study”

**DOI:** 10.1155/joph/9810616

**Published:** 2025-10-13

T. Muto, M. Sakamoto, S. Machida, S. Imaizumi, and T. Sekiryu, “Effect of Vitreous Reflux after Intravitreal Aflibercept Injection for Macular Edema with Branch Retinal Vein Occlusion: A Real-World Study,” *Journal of Ophthalmology* 2024 (2024): 7645490, https://doi.org/10.1155/2024/7645490.

In the article, there are errors in the in-text citations.

In section 2,

• Figure 1a should read Figure 1
• Figure 1b should read Figure 2

In section 3,

“No significant differences were noted in BCVA, RFT, and CFT at the initial visit among the three groups (Figures 3(a) and 3(c); *P* = 0.75, *P* = 0.43, and *P* = 0.48).”

Should read:

“No significant differences were noted in BCVA, RFT, and CFT at the initial visit among the three groups (Figures 3(a)–3(c); *P* = 0.75, *P* = 0.43, and *P* = 0.48).”

We apologize for these errors.

